# Exploring the Potential of Virtual Reality and Artificial Intelligence as Tools to Design, Develop and Deliver Psychotherapeutic Intervention for Family Caregivers of Persons Living with Dementia

**DOI:** 10.1002/alz70858_104542

**Published:** 2025-12-26

**Authors:** Mary Chiu, Adriana Shnall, Amer M. Burhan

**Affiliations:** ^1^ Ontario Shores Centre for Mental Health Sciences, Whitby, ON, Canada; ^2^ Ontario Tech University, Oshawa, ON, Canada; ^3^ Baycrest Centre for Geriatric Care, Toronto, ON, Canada; ^4^ University of Toronto, Toronto, ON, Canada

## Abstract

Our presentation explores the potential of Virtual Reality (VR) and Artificial Intelligence (AI) as tools used in designing, developing and delivering psychotherapeutic interventions for family caregivers (CGs) of persons living with dementia (PwD). Family CGs experience significant emotional burden and strain from unresolved relational issues, which are compounded by the evolving nature of their caregiving roles. While traditional psychotherapeutic interventions have proven effective in reducing CGs distress while enhancing their coping strategies and resilience (Sadavoy et al., 2022), technology‐based solutions may augment these efforts.

VR, particularly through high‐fidelity environments and scripted avatar dialogues, allows CGs to engage in simulated caregiving scenarios, and to practice communication skills and emotional regulation, and deepen their understanding of PwD's experiences, allowing CGs to build empathy. For example, VR‐SIM Carers www.vrsimcarers.ca, a simulation‐based experiential learning platform in VR, allows CGs to “walk through” challenging caregiving scenarios by selecting their responses, while receiving feedback from a virtual clinician and a virtual simulated‐PwD. This safe, self‐paced learning environment offers CGs a space to acknowledge and process their emotions, and to practice communication and coping strategies. AI‐powered tools e.g. real‐time AI responses may also be integrated into VR, to enhance accessibility, recommend personalized strategies tailored to CGs’ situations.

Despite aforementioned advances and benefits, the use of technology in psychotherapeutic interventions raises concerns, particularly regarding the emotional complexities of the CG‐PwD relationship. Long‐standing relational dynamics, including unresolved grief or resentment, can impact caregiving interactions. While AI shows promise in providing compassionate responses (Ovsyannikova et al, 2025), it may fall short of addressing the emotional nuanaces arising from these relationships. Particularly, its limited ability to interpret non‐verbal cues and complex emotional nuances limits its effectiveness in deeply relational contexts.

While technological innovations hold promise for enhancing the mental and emotional well‐being of dementia caregivers, their design must be sensitive to the unique psychological and relational complexities inherent in caregiving. A collaborative, cross‐sector approach that integrates the lived experiences of CGs is essential to ensure these tools are accessible, feasible, and culturally sensitive to the diverse populations they aim to support (Chiu & Saragosa, 2024).

